# Supplementary material for: Start small: A model for tissue-wide planar cell polarity without morphogens
Source: PLoS Comput Biol. 2026 Feb 12;22(2):e1013938. doi: 10.1371/journal.pcbi.1013938 (PMC12928585; doi:10.1371/journal.pcbi.1013938)
Supplement: S1 Table — This table lists all parameters used in the simulations, including volume constraints, neighbour order for lattice site copy events and contact energy calculations and volume fractions of each cell and cell compartments inside the cell. (PDF) [file pcbi.1013938.s017.pdf]

Table S1: **Simulation Parameters:** This table lists all parameters used in the simulations, including volume constraints, neighbour order for lattice site copy events and contact energy calculations and volume fractions of each cell and cell compartments inside the cell.

**Parameter**	**Name**	**Value**
T	CPM fluctuation amplitude	15
n_{copy}	Neighbor range for lattice site copy attempts	3
n_{contact}	Neighbor range for contact energy calculations	4
t	Total time (depends on the case examined)	10^5 to 5×10^7 MCS
cd	Cell Diameter	12
tV	Target Volume	144
λ_{v}	Strength of Volume Constraint	12
	**Volume Fractions**	
p_{P}	Proximal Domain	0.15
p_{D}	Distal Domain	0.15
p_{C}	Cytoplasmic Domain	0.5
p_{L}	Lateral Domain	0.2
	**Cell Proliferation Parameters**	
growth_rate	growth rate for proliferation	0.001
t_{relax}	relaxation time for cell proliferation	varies from 0 to 10^7 MCS for uniform cell proliferation
