# Supplementary material for: Start small: A model for tissue-wide planar cell polarity without morphogens
Source: PLoS Comput Biol. 2026 Feb 12;22(2):e1013938. doi: 10.1371/journal.pcbi.1013938 (PMC12928585; doi:10.1371/journal.pcbi.1013938)
Supplement: Attachment — Submitted filename: Thayambath PCP morphogen report.pdf [file pcbi.1013938.s024.pdf]

This manuscript, "Start Small: A Model for Tissue-wide Planar Cell Polarity without Morphogens," presents a compelling computational study on a fundamental and timely question in developmental biology. The authors investigate whether long-range planar cell polarity (PCP) can be established in a tissue without relying on a global morphogen gradient, a long-held paradigm that has been challenged by recent experimental findings. The work is well-contextualized within the existing literature, and the conclusions are both interesting and significant for the field. The overall approach is solid, but a major revision is required to strengthen the manuscript and its conclusion: expanding the discussion to be more self-critical, improving the presentation of the dynamic simulation results by providing supplementary movie.

Major Strengths:

- **Important and Well-Posed Question:** The central question of how large-scale tissue polarity is robustly achieved is of great importance. The authors frame their investigation around recent experimental results that cast doubt on the necessity of Wnt gradients, making this work highly relevant.
- **Thorough Contextualization:** The introduction provides a comprehensive overview of the molecular mechanisms of PCP and thoroughly reviews the literature on the search for global cues. This effectively establishes the knowledge gap that the study aims to address.
- **Solid and Validated Approach:** The use of the Cellular Potts Model (CPM) is well-suited for this problem, as it allows for the simulation of dynamic cell shapes, rearrangements, and growth. The model, which is based on differential adhesion of subcellular compartments, successfully reproduces key experimental observations, such as the domineering non-autonomy of Fz and Vang mutant clones. This validation lends credibility to the model's core mechanics.
- **Interesting and Plausible Conclusion:** The main finding is that a combination of a local boundary signal, a small initial tissue size, and subsequent uniform cell proliferation is sufficient to establish robust, long-range polarity. This provides a plausible, morphogen-independent mechanism for establishment of tissue polarity.

Major comments :

1. **Expansion of the Discussion Section:** The current discussion

accurately summarizes the findings but is somewhat limited. It would benefit from a more critical and forward-looking perspective. Furthermore, a division into subsections with summarizing titles would give the discussion more structure, particularly after the proposed expansion.

- The authors should discuss the limitations of their approach. For instance, how sensitive are the results to the specific contact energy parameters chosen? What are the advantages or disadvantages of using a differential adhesion model compared to more traditional reaction-diffusion equations to represent intracellular protein dynamics? Furthermore, the central premise relies on the tissue "starting small." The authors should discuss how this model reconciles with biological systems where tissues may already be large (many more than 8x8 cells) before PCP is established.
- The authors claim at several occurrences that the mechanism they propose is robust, but they should prove that point convincingly. They should also discuss what is really new in their work. And whether the timescales are more realistic than in Ref. 37.
- The manuscript concludes by briefly mentioning that the framework could be used to study other developmental processes like convergent-extension. This section should be expanded. The authors could elaborate on *how* this model could be adapted to investigate these phenomena and what specific open questions it could help answer.

2 **Enhanced Presentation of Dynamic Data:** The processes being modeled (cell growth, division, and the emergence of polarity) are inherently dynamic.

- **Supplementary Movies:** Static figures, while useful, cannot fully capture the evolution of the system over time. Providing supplementary movies for the key simulations (e.g., the emergence of swirls in large tissues, the propagation of polarity during uniform growth vs. front-only growth) would be immensely valuable for the reader to develop an intuition for the model's behavior.
- **Figure Clarity:** Some figures could be improved for clarity. For example, in Figure 2A, the use of curved arrows to denote periodic boundary conditions is not immediately intuitive. The choice of colors in the mutant analysis (Figure

2E, E') is also slightly confusing, as the mutant cells (dark red/green) are meant to represent the loss of function of the proteins represented by magenta and green in the model schematics.

Others :

P. 1, several occurrences of the word "epithelial": this is relevant for fly, but PCP is also important for morphogenesis of mesenchymal tissues in mouse, human, xenopus (see e.g. Wallingford et al., Nature, 2000, 10.1038/35011077).

P. 1, bottom: clarify § "The core pathway...", it could benefit from a diagram

P. 2: § "Experiments and mathematical model" and § "Wnt lignands" make a long discussion about potential global biochemical gradient, but it is not clear what point the authors are trying to make. They should indicate here (rather than later) that other mechanisms might be at work and are worth exploring.

P. 3 « *We implement the mutual exclusion of these compartments inside cells and intercellular signalling using the differential adhesion properties of these compartments.* »: Justify why intracellular protein-protein interactions can be modelled with differential adhesion properties.

P. 4 Eq 4: Choosing a constant cell-cell junction tension (rather than a variable one, which is more realistic) is a choice which is acceptable for the sake of simplification, but that should be explicitly mentioned and justified.

P. 5 « *We determined the exact values for each contact energy term by extensive parameter scans aimed at optimizing polarization for small systems.* »: It would be interesting to discuss the sensitivity of these parameters. It doesn't require a full analysis, but at least a qualitative discussion from experience which would help the reader to develop an intuition.

P. 5 Eqs 5-6: Both the average of modulus and the modulus of average are interesting in the context of PCP and could be discussed. The authors seem to be interested only in the modulus of the average, which

makes sense (I find that interesting in the context of the present question regarding long range ordering). However, according to Eq. 6; they seem to have measured the average of the modulus which makes sense for local ordering. Please clarify and justify.

P. 7 Fig. 2: There are too many arrows with different (and unclear) meanings. Suppress most of them, keep only the necessary ones, and provide a clear caption

P. 7 Fig.2 (and Supp Fig. 1): The choice of colors should be consistent. If subdomains are indicated by pink and light green, while schematics use either red-brown and dark green, it creates a confusion. Mutant cells could rather be evidenced using transparency.

Up to P. 8 included the results are mostly expected ones. In particular: « *From these results, we conclude that the presence of the boundary signal in our model successfully biases the system to polarize along a preferred direction.* » Is this something that other models struggled with?

P. 9 Fig. 3E caption: clarify « local polarization as a function of radial distance »

P. 10, top: clarify and rewrite § "For the periodic" and § "We also examined".
2 typos: polalized, distancesup

P. 11 Fig. 4D: caption typo inidicated
Fig 4HIJ: add zooms of the cell and subcell compartments (possibly in the Supp)

P. 12, § "Instead": this is the main result of the paper, it is non-trivial, it should be more developed.

P. 12 Discussion: remove all redundancies with Introduction.

P. 13: Do daughter cells inherit PCP in a symmetric or asymmetric ways? Does that depend on the cell elongation?

P. 13 bottom, § "Our results": overclaimed. Support or downtone the claims.
