# Supplementary material for: Start small: A model for tissue-wide planar cell polarity without morphogens
Source: PLoS Comput Biol. 2026 Feb 12;22(2):e1013938. doi: 10.1371/journal.pcbi.1013938 (PMC12928585; doi:10.1371/journal.pcbi.1013938)
Supplement: Attachment — Submitted filename: responses_to_reviewer_comments.pdf [file pcbi.1013938.s025.pdf]

Reviewer #1:

A long-standing question in polarity field is how long-range polarity is established and whether morphogen gradients are required. This paper describes a new computational model of planar cell polarity (PCP) that lacks long-range cues, which have been shown to be required in most other theoretical models of PCP. Using a Cellular Potts framework and modeling the partitioning of proximal-distal compartments using differential adhesion of opposing complexes within and between cells, the authors show that this can give rise to spontaneous polarization with strong local alignment in small tissues. However, larger tissues lack long-range alignment without a directional cue, even when an orientation boundary is provided, giving rise to high local order but little global alignment (swirling patterns), as observed in prior models. Coupling a polarizing boundary in a small field of cells that is allowed to grow with uniform proliferation results in robust large-scale polarization, as daughters cells will repolarize utilizing cues from their neighbors. This work nicely demonstrates that a small tissue adjacent to a polarizing boundary can establish long-range polarization if coupled with cell proliferation, as was previously proposed in vivo in the Drosophila larval wing (Sagner et al, 2012).

Although there are many theoretical models for PCP, the current phenomenological model is distinct in its use of the Cellular Potts framework, and by incorporating growth and proliferation as a mechanism to propagate/expand existing polarization. This is exciting because it points experimentalists searching for the elusive global cue for PCP in a new direction, suggesting that an edge or a boundary, rather than a graded morphogen, may be sufficient especially if polarization is established when the tissue is small. These findings will be of broad interest to those interested in tissue patterning and cell polarity.

I have some comments that would improve the readability of the manuscript.

1. In Figure 2A, the model is described as continuous so that all cells had one junction. But I assume this was not implemented for cases involving a boundary (Fig. 2B) or leading edge expansion. Needs clarification.

In Figure 2A, the cells are arranged on the lattice with periodic boundary conditions. This implies that every cell has the same number of neighbours on average (about 6) and no cell experiences a physical (non-cellular) boundary. In Figure 2B, there is a physical boundary at the left edge of the lattice and an open space on the right edge. So although cells in the first column share an edge with the boundary, they are not treated differently; instead, the boundary is implemented as a static/fixed interface with the same adhesion/contact properties of a 'distal' cell compartment. On the front open boundary, cells are in contact with the Medium, which does not distinguish between proximal and distal compartments. The lateral compartment, however, is less preferred compared to the proximal and distal compartments.

2. Given that a 'distal' orienting bounding is sufficient to generate aligned polarity in small tissues with growth, I wondered what would happen if the polarization boundary was of 'lateral compartment' character. Does this provide any bias to align polarity w/ or w/out cell division? In the mouse skin, polarity initiates from the midline outward (Aw et al 2016).

Thanks for this suggestion! In our model, although the front boundary/medium has no preference for proximal or distal compartments, the lateral compartment has slightly less preference. So we examined the effect of a lateral boundary signal for two different cases: (1) higher preference of Medium for proximal or distal compartments over lateral compartment [$J_{ml} > J_{mp} = J_{md}$], and (2) equal preference of Medium for both proximal, distal and lateral compartments [$J_{ml} = J_{mp} = J_{md}$]. In both the scenarios, we observe that the cells do not align along the proximal-distal axis. Instead each column of cells end up independently choosing between up/down alignment. As a result, the average global polarization

decreases (about 55%) and the mean angle of tissue polarization is biased around +/- 90 degrees (Added as Supplementary Figure S10).

3. Figure 3 shows the model with leading edge divisions is less successful in propagating polarity compared to uniform divisions. I wondered why this condition was tested in the first place. Are there example of tissues that grow in this way? Perhaps during wound repair or limb outgrowth? Other aspects/assumptions of the model (repolarization during division) are rooted in experimental observations, so it would be helpful to place leading edge divisions in a physiological context as well.

Wound repair is a good example of a proliferation happening at the leading edge. Other examples would include bone growth, tissue regeneration and the retina growth cone, for which a PCP model was proposed by Salbreaux et al in 2012.

4. Figure S4 plots the minor versus random axis division impact on polarization in the uniform or leading-edge division models. The minor axis had small (~3%) improvement over the random axis. Firstly, the long axis is never explicitly tested and secondly, I think the statement above is too strong for such small improvement.

We tested the major vs minor axis explicitly and found that for uniform cell proliferation, there is little to no difference in phi as the tissue expands. However, for cell proliferation on front, the global polarization decreases with tissue size for divisions along the major axis. For divisions along the minor axis, although there is an initial dip in polarization, it increases (~1% increase) as the number of cells increases.

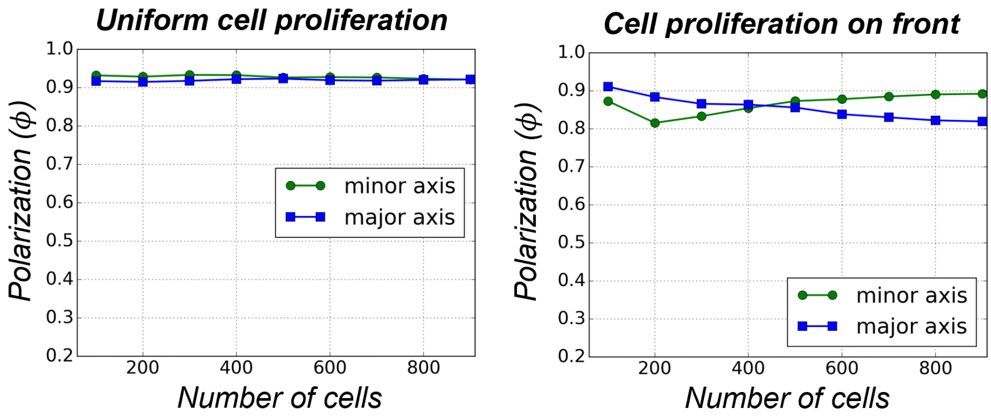

Added as supplementary figure S12.

For cell division for uniform proliferation on front, we plot the time series of global polarization, the number of cells in the system, and the mean angle of tissue polarization (positive and negative angles separately). For minor axis cell divisions, the mean angle deviates from the proximal-distal axis much earlier compared to major axis cell divisions.

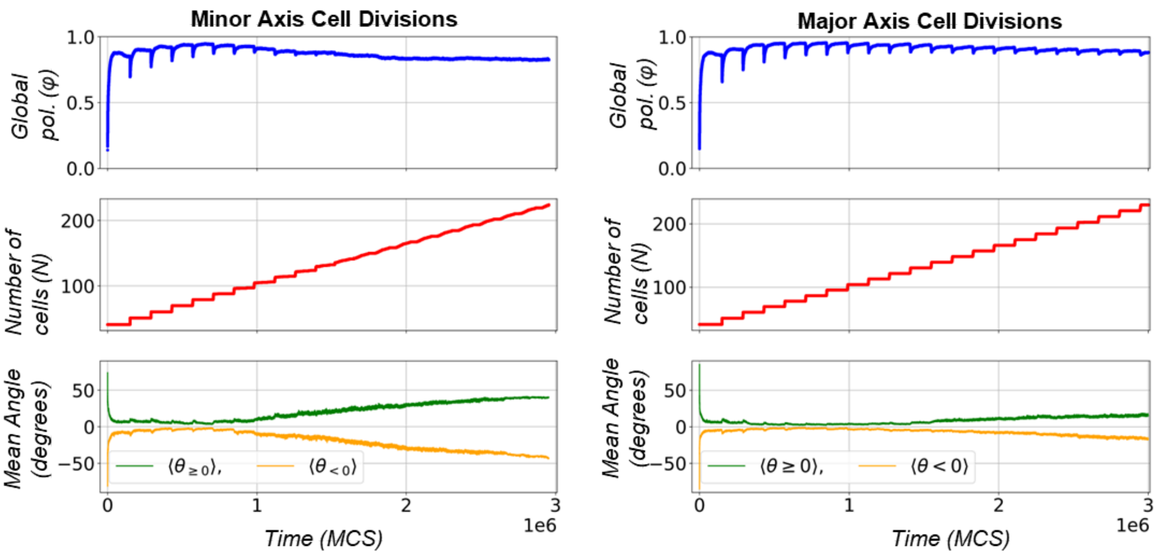

5. Some of the language related to division orientation is confusing to an experimentalist. Division orientation typically refers to spindle orientation i.e., according to Hertwig's rule, cells divide with the spindle aligned with the cell's long axis. The cleavage plane is therefore aligned with the short/minor axis. In the discussion, "division along the long axis results in daughter cells maintaining more surface contact with each other than with external neighbours, reducing their access to external polarity cues and leading to weaker polarization." I think if the authors use the terms 'spindle alignment' and 'cleavage plane' to describe their data, this will be more accessible to experimentalists.

We clarified this in supplementary figure S12 A. The cleavage plane is what we refer to as major/minor axes of cells.

6. In the discussion, the authors explain why they chose model parameters that allow cell autonomous polarization, but state that other parameters that prevent cell-autonomous polarization are also effective in generating long-range polarization. It would be useful to include these data as a supplementary figure.

We thank the reviewer for this suggestion. We further explored the cell non-autonomous model and found very interesting results. We decided to expand the scope of our paper and added a new section in our results to discuss the differences between those models together with a new main figure (Fig 5) and Supplementary Figures S7 and S8.

7. Figure S1B/C, S5 and TableS1 are not referred to in the text.

Figure S1 B panel left is referred to in the text (in Model Overview section). The panel right of S1 B is removed now. Figure S1C is now referred to in the section of the effect of system size on global and local polarization. Figure S5 is now renamed as S11 and is referred to in the Discussion section. Table S1 is now referred to in the Model Overview section.

Reviewer #2:

The paper revisits the self-organization problem of Planar cell polarity, PCP, the alignment of cell polarity along epithelial sheets that underlies several morphodynamic processes in vertebrates. Whereas several

key molecular pathways have been uncovered over the years and several computational models have shown that local cell-cell interactions can explain self-organization on a local scale, most models to date have required a global cue to explain patterning across large tissues. However, experimental efforts to uncover said global cue have not succeeded so far, raising the question if such a global cue truly exists.

In this work, the authors build a cellular Potts model in which PCP emerges without the need for a global polarity cue, through a combination of local symmetry breaking followed by symmetry maintenance during a proliferative process.

The model is nice because it is the first to show spontaneous emergence of PCP in larger systems without the presence of a global cue, and several interesting analyses are performed throughout the paper. As such, the paper has the potential to be a valuable contribution to the field. However, I do have some open questions that I think should be addressed before publication.

QUESTIONS / COMMENTS :

(1) It was not immediately clear to me what exactly the motivation for some of the experiments and the overall work was. This actually became much clearer when reading the discussion:

1a) for example on P12 : "*While the mechanisms of local planar cell polarity …. All these negative results suggest that an alternative mechanism, independent of morphogen gradients, may be required to establish global PCP orientation*." I feel like this captures the essence of the paper quite clearly and concisely – but for me, this message got a bit lost in the introduction, likely because of the amount of detail given about the underlying molecular mechanisms (details which do not seem to be used throughout the paper. My suggestion would be to swap these parts: the discussion paragraph above is ideal for the problem statement in the introduction because it is quite conceptual. The discussion could then go into more detail about how this now ties in with known molecular mechanisms and what it might mean for that field.

Thanks for this suggestion. We moved the first paragraph of the Discussion section to the Introduction and merged it with the old first paragraph.

1b) The same holds for the motivation for the experiments with different boundary conditions in Fig2, as it was not clear to me what the biological motivation for these experiments was – until the discussion on p12 "*Sanger et al proposed that PCP may be established early in development, while the tissue is still small, with the guidance of local boundary signals, and long-range tissue orientation arises through subsequent cell proliferation*". This motivation should ideally provided in the introduction and/or in the results section where these experiments are performed.

Following this and other reviewer comments, we completely rewrote the Discussion section and streamlined the Introduction to address this and other concerns.

1c) The discussion nicely places the results in context of literature. But I would also like to see some ideas on how this model could now be experimentally tested, and how (if at all) it differs in behavior from existing models with global polarity cue. It is nice that this model explains the data without the need for this global polarity cue, but that still does not guarantee that this is the "correct" model. That is fine, but it would be good if the discussion can provide further directions on how to continue from here.

We added a dedicated paragraph in the Discussion to talk about how our model could be tested experimentally. We cannot go into the specifics about the experiments, of course, but we gave some general guidelines of possible ways to test our model predictions.

(2) Not all parameter values are described: what is T? What is lambda_V? What is V_t? What is the field size? To ensure the work is reproducible, these details should be added.

*T, lambda_V and V_t are defined in the Model Overview section. The actual values of the parameters used in the Model are given in Supplementary Table S1. The dimensions of the lattice are defined based on the number of cells in the system. For example, the cell diameter that we use is 12 lattice sites. So, for a system containing 4x4 cells, the lattice dimensions are 4*cell diameter x 4*cell diameter = 48 x 48 lattice sites.*

(3) P8 "*We found that as the number of cells increases, global polarization decreases in both configurations (Fig 3A)*." and P10 "*This clearly suggests that when the system size increases, there is no long-range order across the entire system in the absence of a directional cue*".

3a) I am not sure this is fair since the time it takes to reach the ordered state also increases (maybe even exponentially?) with system size. From Fig S1C it is clear that the larger systems have not yet plateaued at the maximum simulation time. So from this it is not clear whether it is fundamentally impossible to achieve the global ordering, or that it just takes (much) longer. I think it would be useful to complement this analysis with "time taken until a certain amount of ordering is reached" – e.g. the time it takes to reach phi = 0.5, to give you a sense of how the required time scales with system size.

For uniform cell proliferation with a refractory time of 10^4 MCS, starting from 40 cells, the system reaches 900 cells after roughly five divisions, which happens at around 10^6 MCS. At that stage the system has kept a stable global polarization at around phi=0.9. In contrast, without proliferation, the final global polarization after 10^6 MCS is only phi=~0.5 for both periodic and left-boundary signal configurations in a square geometry (30x30 cells), and only phi=~0.3 for the configurations with left boundary and 10x90 cells. Although it is possible that non-proliferating systems could eventually reach higher global polarization values given enough time, proliferation offers a much more efficient and fast strategy.

At the suggestion of the referee, we computed the time to reach 50% polarization for those different cases (Supplemental Figure S8) from where we can see how those times increase with system size.

3b) Related: it would be useful to give some indication of how the CPM time in MCS could be related to real time, if possible. For example, is it known how fast individual cells can reorient, and could you use this to set/estimate this scaling from Fig S1A? And if you do, could you compare the predicted ordering time to some experimental data? (Not perfectly, just in terms of order of magnitude to begin with)? Likewise , is the doubling time then realistic?

Thanks for this question. We can in principle use the time that simulated cells take to polarize by their own (for the cell autonomous version) or the time a single cell reorients within a tissue as the time unit of our simulations. However, it is not clear if these are measurements that can be easily performed in experimental systems. We could not find any data with these measurements in the literature so we chose to keep the standard model time units (MCS) and use it as a way to compare the results of the different model versions in a qualitative way.

(4) The paper occasionally seems to conflate two concepts: emergence of order from disorder, and

maintenance of order that is already there. For example P10 "*This suggests that the distance over which the polarity alignment is maintained, the correlation length, is very small*" and "*This indicates that the boundary signal helps maintain the cell polarity*". But these experiments all focus on emergence of the order from an unordered system. Since the time to reach the ordered state also increases rapidly with system size (Fig S1C), this does not allow you to ask the second interesting question: what is the max amount of ordering the system can maintain? For that, I think it would be interesting to do a complementary experiment where the cells are initialized in the ordered state, and you evaluate over what spatial scale this ordering is maintained over time.

Thanks for that comment and suggestion. We performed simulations for fixed tissues of variable size that already start from a perfectly polarized initial condition and noticed that their degree of polarization decreases linearly over time, with a higher rate for larger systems. We then compared this with equivalent simulations where the cells start at a disordered state. While we expect that two systems will eventually stabilize their polarity at similar values, we noticed that the loss of polarization happens at a slower rate than the gain of polarization for smaller systems, but the rates seem to be comparable for larger systems. (added as Supplementary Figure S14). We also found that the rate at which cells maintain their initial order also depends on the packing geometry of cells in the initial configuration (Figure S15). If the cells are perfectly packed in an hexagonal arrangement tissue polarization is maintained as time progresses. The tissue gradually loses polarization only when the initial packing is irregular.

(5) P5 "*We determined the exact values for each contact energy term by extensive parameter scans aimed at optimizing polarization for small systems.*" It would be good if the authors could show the results of that parameter scan. How wide/narrow is the regime where this polarization occurs? Are results in the paper expected to hold across different local polarization parameter scenarios?

Added as Supplementary Figure S2.

(6) P8 "*The addition of this boundary signal not only biases the global alignment direction but also enhances the global polarization compared to the periodic boundary configuration for nearly all time steps in the simulation (Figure 2D)*".

6a) Could this be because the leftmost column of cells has a different shape and (potentially) a less variable, high phi? I think it would be worthwhile to check if this still holds when only considering the middle two columns of cells, which are more comparable in shape between the two scenarios.

Indeed the left-most column of cells has a slightly different shape due to the straightness of the left boundary, but note that on their right side the cells already have a naturally shaped boundary with the second column of cells. So, even if the first cells had an unnatural higher alignment due to their shape, we could still treat them as our effective signal boundary and the results from the analysis of the remaining tissue (second column onwards) would yield the same results.

6b) Related: there is of course some anisotropy because this is a lattice-based model, which also has implications for the sides of the (hexagonal) cells. Does the left boundary signal scenario in Fig 2B still give the same 4 outputs as in Fig 2A? To what extent is the distribution of order parameter values (say at the final timestep) similar between the 4 potential output directions shown in Fig 2A? If you put the boundary at an angle, do the results still hold?

The left boundary signal scenario biases the global polarization direction to the proximal-distal axis (to the right), which can be seen in our radial plots (Fig 2C). However, the degree at which this is biased depends on the system size. For smaller systems, the mean angle of tissue polarization is predominantly along the proximal-distal axis (ref Fig 2C panel right). For larger systems, the first few columns of cells are aligned

along the proximal-distal axis, however global alignment is lost as we move further away from the left boundary. As a result, the mean angle is more dispersed about the proximal-distal axis (Fig 3B right panel).

To study the effect of a boundary at an angle, we created a system where we varied the angle between the boundary and the y-axis. To discard boundary effects we increased our system size in the vertical direction and calculated the global polarization only for the cells located near the center of the lattice (as indicated by the red box in the figure below). As expected, the tissue polarizes mostly along the direction orthogonal to the boundary, with small deviations for intermediate angles between 0 and 45 degrees, indicating some minor lattice grid effects. From these we conclude that the observed anisotropy in tissue polarization is mainly due to the presence of the left boundary and not to the lattice.

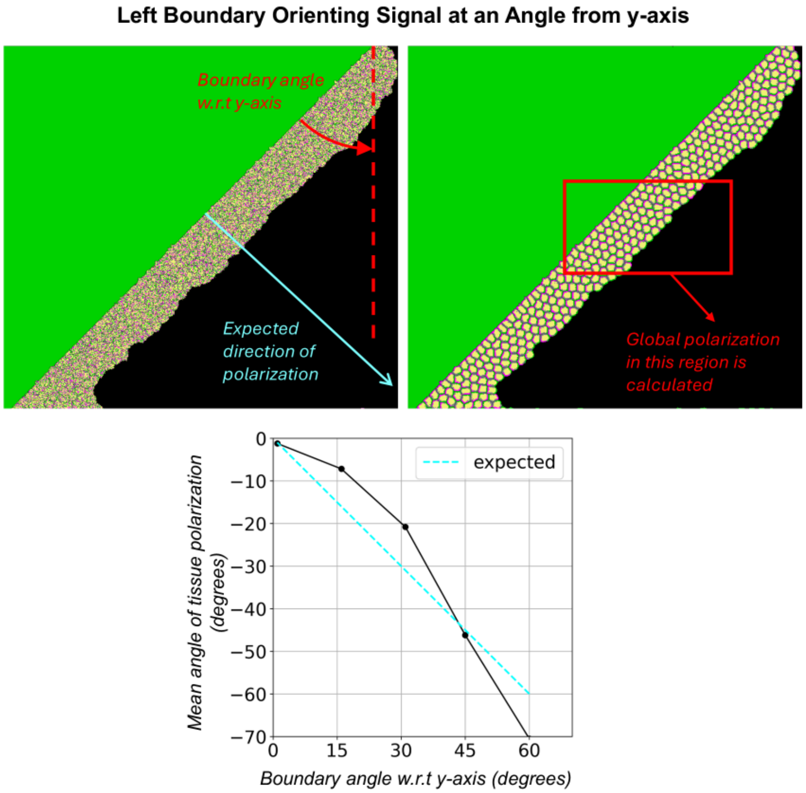

(7) P12 "*we observe that the alignment is not strictly along the x-axis but instead shifts towards +/- 60 degrees*"

7a) I find this somewhat suspicious because this would correspond to the sides of the hexagonal cell shape. Is there any way to check if this is a "real" result or if anisotropy is at play here? What if you placed the boundary signal at an angle?

There could be effects of anisotropy of the lattice, but it is hard for us to rule that out. From the simulations we mentioned in answer 6b, where we varied the boundary angle, we would expect a pinning along the ±45° angle direction, so the cause for the 60° direction alignment seems to lie elsewhere. We believe that this might be caused by the space geometry and the resulting packing arrangements of the cells, which tend to align into vertical rows. CompuCell3D only supports rectangular and hexagonal lattices, and when we repeated those simulations with the hex grid we found the same ±60° deviation.

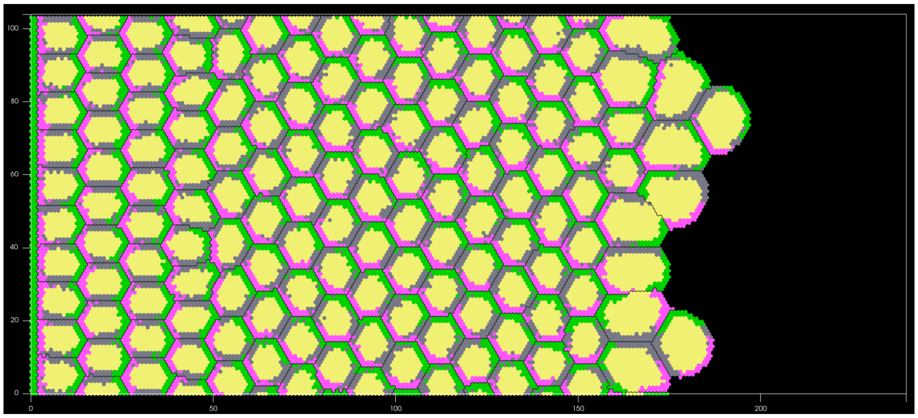

We also note that the choice of cell division axis has an effect on how soon this happens on the distal-front proliferation model. Comparing the time series data of the evolution of mean angle of tissue polarization for the two cases (see Supplementary Fig S4) revealed that divisions along the minor axis exhibited greater misalignment around ±60° early on compared to divisions along the major axis. In minor-axis divisions, the daughter cells facing the distal boundary lack contact with the aligned cells at the bulk of tissue and are more prone to failure in reestablishing their polarity, whereas in major-axis divisions, both daughter cells maintain contact with the bulk tissue after division. The loss of such positional information could explain the increased misalignment observed in the minor-axis case.

7b) Related on p13 "*We believe that the directional shift in polarity alignment observed for cell proliferation on the front scenario could be due to the lack of ability of the newly divided cells at the boundary to fully integrate polarity cues from all directions*". Interesting hypothesis, but could you test this further with some exps/further analysis? E.g. testing how cells polarize when surrounded by varying numbers of cells, but you may be able to come up with other experiments as well.

See the response above regarding major and minor axis divisions in cell proliferation on front (see Supplementary Fig S12E left panel, Fig S4)

In addition to this, we did some experiments with a smaller system under two proliferation scenarios as suggested by the reviewer. Starting with a group of 4x9 cells that is already polarized along the proximal-distal axis, we allowed (1) the first column of cells facing the open boundary to grow and divide ***once****, (2) the fifth column of cells (fully surrounded by other cells) to grow and divide* ***once.***

We then calculated the time taken for these cells to polarize along the proximal-distal axis right after they divided. This was done for an ensemble of 100 simulations and the average is plotted below for (1) growth/division on distal front and (2) growth/division inside. Our results show that cells on the front take much longer to align with the rest of the tissue compared to cells that divide inside the tissue.

Average Time taken for Dividing Cells to Re-polarize along Proximal-Distal axis

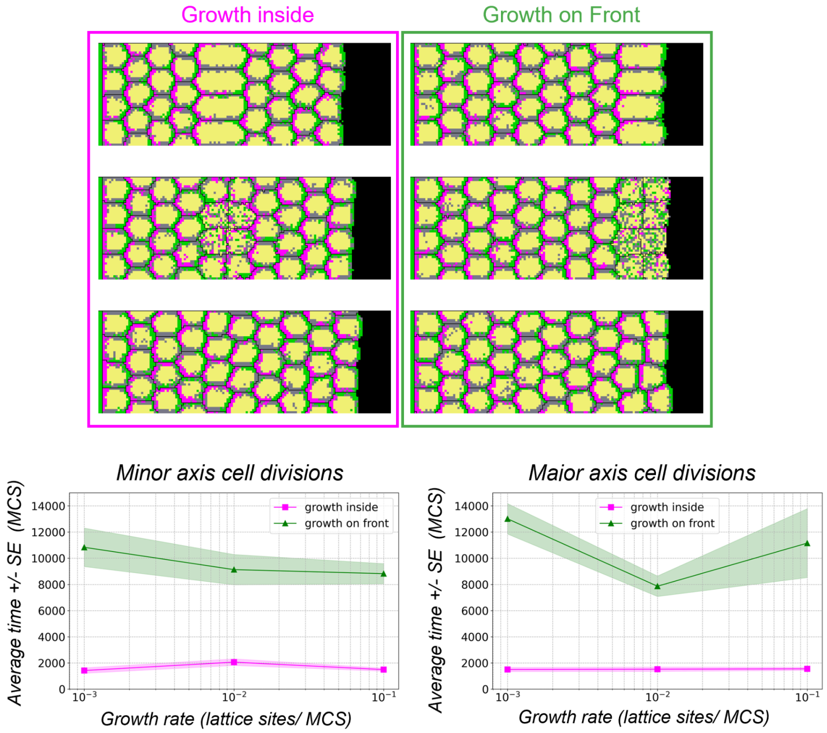

(8) P12 "*Results remain the same for slower growth rates, but polarization is gradually lost for faster growth rates, suggesting that cells need some time to adjust their polarity before the next round of division to maintain local tissue alignment (Fig S6)*". Could you further support this hypothesis by comparing the distribution of "time between divisions" to that of "time taken by a single cell to (re)polarize"?

We analyzed the time taken for a single cell to repolarize as a function of growth rate when the cell either divides at the front or in the bulk of the tissue. To our surprise we found no clear effect of growth rate on the time a single cell takes to polarize after division. However, we do note a clear effect of the refractory time (the time taken for a cell to resume growth after division) on the tissue polarization (Fig S11), which supports our interpretation. We believe that this is a collective effect - increasing growth rates effectively decreases the time between division events the same way as decreasing refractory times. Since cells lose polarity after division, every cell division event has a disrupting effect on the neighbors, so an increasing rate of divisions is akin to an increase in the "effective temperature" of the system that negatively affects the maintenance of polarization order.

(9) P13 "*We chose our model parameters (see Table 2) to reflect the latter case; however, setting the internal contact energies to negative values to prevent cell-autonomous polarization still allows spontaneous tissue polarization, and the key results from our model remain qualitatively the same*". That is nice to hear – but ideally, these results should also be shown in the paper and/or supplement.

Another reviewer had the same suggestion. We further explored the cell non-autonomous model and found very interesting results. We decided to expand the scope of our paper and added a new section in our results to discuss the differences between those models together with a new main figure (Fig 5) and Supplementary Figures S7 and S8.

MINOR COMMENTS TO THE AUTHOR (no response required):

- P3 “*However, all these models are based either on a pre-established PCP order from which the applied tension realigns the orientation or on a specific sequence of PCP activation of cell rows that facilitates global tissue alignment*”. What is meant by “sequence of PCP activation of cell rows”? Please clarify.

We are referring to the model by Salbreaux et al (https://doi.org/10.1371/journal.pcbi.1002618) where different rows of cells are allowed to polarize at different times in a sequential manner from proximal-most row to distal-most row.

- Tables 1 and 2: does this imply that the adhesion energies are asymmetric, or do you simply report as a diagonal matrix to avoid duplicate values? Please clarify.

The energies are symmetric with respect to proximal-distal compartments (J_pp = J_dd, J_lp = J_ld, J_mp = J_md). We do not report the diagonal values to avoid duplication.

- P3 “*Within each cell, these compartments exhibit low affinity for each other, leading to their spatial segregation*” – I think in the CPM this would be better described as “actively repel” each other than “have low affinity”, as the latter (neutral adhesion values) would probably not lead to segregation.

The contact preferences in CPM comes from the minimization of the energy term where contact energies are used. Because this depends on the relative values of contact energies between different domains and not on a specific value threshold that defines repulsion or attraction, we chose to use the relative terms “low affinity” for high contract energies, and “high affinity” for low contact energies between domains.

- P4 “*formed by a collection of lattice sites on a square or hexagonal grid in 2D or 3D*”. In this work, I assume it is only 2D square? If yes, this should perhaps be mentioned here.

Removed the word hexagonal from the manuscript since we don’t use hexagonal lattice for the model. We also removed the word 3D as all our models here are two dimensional.

- Fig 1 phi = 0, phi = 1 – I assume these are not the measured values? Consider replacing either with the measured values or phi ~ 0, phi ~ 1 (i.e. replace "=" with "approximate =")

Done.

- Fig 1B’ what does the blue box mean?

The blue box serves as a guide to highlight the evolution of a cell during the simulation. We added that information to the legend.

- Fig 2A what does the red box mean?

This is to indicate a correspondence with the angle distribution plotted in the middle panel of Fig 2C. We added that information to the figure legend to make this clearer.

- Fig 2C is this the distribution of individual cell angles within a simulation, or of mean cell angles between the 100 simulations?

Individual cell angles in a simulation. We changed the figure and legend to make this clearer.

- Fig 2D can you give an indication of the robustness of this order parameter across simulations? Plot shows the mean, but what is the SD/standard error?

Modified the FIg 2D. Added SE along with mean values of global polarization. Also increased the total time to 10^7 MCS.

- What boundary conditions were used in Figs 2E/2E'?

We used the left boundary signal to first align all the cells along the proximal-distal axis. After that we removed the boundary orienting signal and created the mutant cells.

- Fig 2A,2B: text refers to "a system of 8 x 8 cells" but the figures show 4 x 4.

4x4 snapshots were just for visualization.

Reviewer #3:

This manuscript, "Start Small: A Model for Tissue-wide Planar Cell Polarity without Morphogens," presents a compelling computational study on a fundamental and timely question in developmental biology. The authors investigate whether long-range planar cell polarity (PCP) can be established in a tissue without relying on a global morphogen gradient, a long-held paradigm that has been challenged by recent experimental findings. The work is well-contextualized within the existing literature, and the conclusions are both interesting and significant for the field. The overall approach is solid, but a major revision is required to strengthen the manuscript and its conclusion: expanding the discussion to be more self-critical, improving the presentation of the dynamic simulation results by providing supplementary movie.

Major Strengths:

• Important and Well-Posed Question: The central question of how large-scale tissue polarity is robustly achieved is of great importance. The authors frame their investigation around recent experimental results that cast doubt on the necessity of Wnt gradients, making this work highly relevant.

• Thorough Contextualization: The introduction provides a comprehensive overview of the molecular mechanisms of PCP and thoroughly reviews the literature on the search for global cues. This effectively establishes the knowledge gap that the study aims to address.

• Solid and Validated Approach: The use of the Cellular Potts Model (CPM) is well-suited for this problem, as it allows for the simulation of dynamic cell shapes, rearrangements, and growth. The model, which is based on differential adhesion of subcellular compartments, successfully reproduces key experimental observations, such as the domineering non-autonomy of Fz and Vang mutant clones. This validation lends credibility to the model's core mechanics.

• Interesting and Plausible Conclusion: The main finding is that a combination of a local boundary signal, a small initial tissue size, and subsequent uniform cell proliferation is sufficient to establish robust, long-range polarity. This provides a plausible, morphogen-independent mechanism for establishment of tissue polarity.

We thank the reviewer for their appreciation of our manuscript strengths.

Major comments :

1 Expansion of the Discussion Section: The current discussion accurately summarizes the findings but is somewhat limited. It would benefit from a more critical and forward-looking perspective. Furthermore, a division into subsections with summarizing titles would give the discussion more structure, particularly after the proposed expansion.

◦ The authors should discuss the limitations of their approach. For instance, how sensitive are the results to the specific contact energy parameters chosen?

Following a similar comment from another reviewer we added the results from our parameter scans in the supplementary (Fig S2) that shows the wide range of values where the model still works. We mention that in our Model Overview section.

What are the advantages or disadvantages of using a differential adhesion model compared to more traditional reaction-diffusion equations to represent intracellular protein dynamics?

Advantages: we can explicitly model cell depolarization and repolarization. Alignment emerges from local cell interactions. The model is stochastic. Cells are explicitly represented and can easily change shape. Ability to model cell growth and division without the use of hybrid models (vertex models + reaction diffusion). Ability to couple this model with different cell processes downstream of PCP. We added this to our revised Discussion section.

Furthermore, the central premise relies on the tissue "starting small." The authors should discuss how this model reconciles with biological systems where tissues may already be large (many more than 8x8 cells) before PCP is established.

Our model shows which elements are necessary to guarantee that a tissue achieves long-range polarity without the aid of global morphogens, but makes no claim that this is the only way to achieve that outcome. A few alternatives would be (1) the use of a double boundary signal (at the proximal and distal sides) that will double the size that a tissue can align without any growth process, and (2) a cascade of PCP activation starting from a signaling boundary. We now discuss that in the paper. We added this to our revised discussion section and as Supplementary Figures (Fig S9, S16).

◦ The authors claim at several occurrences that the mechanism they propose is robust, but they should prove that point convincingly. They should also discuss what is really new in their work. And whether the timescales are more realistic than in Ref. 37.

We refer the reviewer to our previous answer about time-scales. Unlike the model by Burak & Shraiman, where they can match time units using the diffusion rate of proteins, in our model we would need the time for cell to polarize (or repolarize) to establish the MCS to second conversion, but we could not find that measurement in the literature.

As for the robustness of the mechanism, figure 4E shows that global polarization is maintained for a long time even in the presence of an increasing number of cells. While we did not extend our simulations past 1000 cells, we did not observe any decrease in phi for those higher counts. We now discuss this in the manuscript.

◦ The manuscript concludes by briefly mentioning that the framework could be used to study other developmental processes like convergent-extension. This section should be expanded. The authors could elaborate on how this model could be adapted to investigate these phenomena and what specific open questions it could help answer.

We are preparing a whole methods paper about the subCellular Potts technique. Unfortunately we were not able to submit that manuscript concurrently with this one, as we initially planned, but in the figures below you can see a wide range of applications where our PCP model is tied with other cellular processes:

PCP mediated Cell Elongation

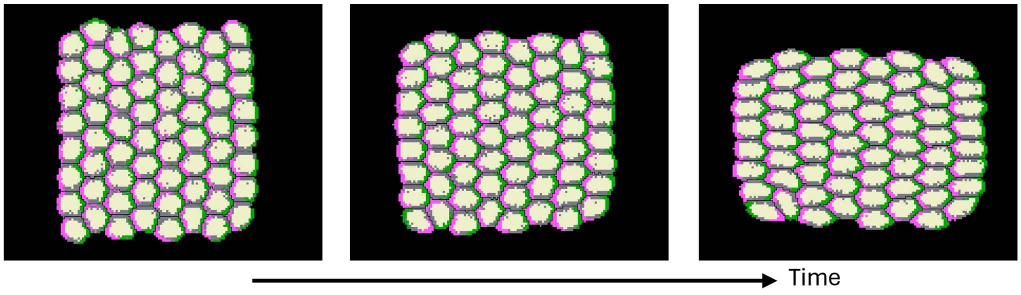

PCP mediated Cell Migration

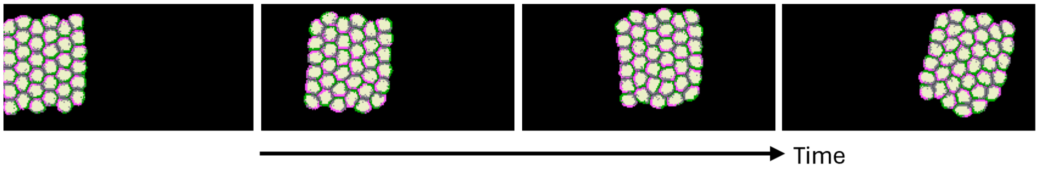

Medio-lateral Intercalation in Gastrulation/ Primitive Streak Formation

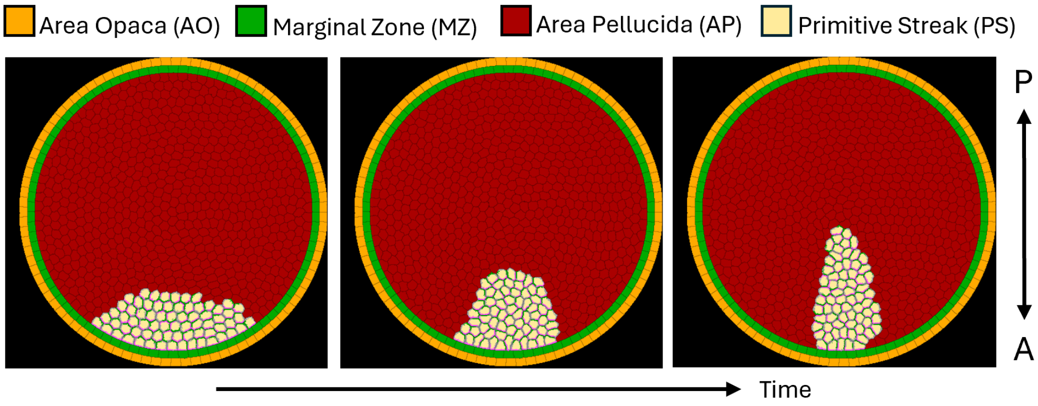

2 Enhanced Presentation of Dynamic Data: The processes being modeled (cell growth, division, and the emergence of polarity) are inherently dynamic.

◦ Supplementary Movies: Static figures, while useful, cannot fully capture the evolution of the system over time. Providing supplementary movies for the key simulations (e.g., the emergence of swirls in large tissues, the propagation of polarity during uniform growth vs. front-only growth) would be immensely valuable for the reader to develop an intuition for the model's behavior.

We added supplemental movies to this resubmission. The movies correspond to different boundary conditions (periodic and left boundary signal) for small and large system sizes along with the cell proliferation on front and uniform proliferation scenarios.

◦ Figure Clarity: Some figures could be improved for clarity. For example, in Figure 2A, the use of curved arrows to denote periodic boundary conditions is not immediately intuitive. The choice of colors in the mutant analysis (Figure 2E, E') is also slightly confusing, as the mutant cells (dark red/green) are meant to represent the loss of function of the proteins represented by magenta and green in the model schematics.

We have changed our figures and color schemes to improve clarity.

Others :

P. 1, several occurrences of the word "epithelial": this is relevant for fly, but PCP is also important for morphogenesis of mesenchymal tissues in mouse, human, xenopus (see e.g. Wallingford et al., Nature, 2000, 10.1038/35011077).

That is an important point. We added that information to our manuscript to highlight the breadth of situations where PCP occurs.

P. 1, bottom: clarify § "The core pathway...", it could benefit from a Diagram

Since our model is agnostic to the exact details about the molecular mechanisms during PCP signaling we prefer to not show them and refer the readers to other papers where this is discussed in more detail or where the models rely more explicitly in the specific implementation of those pathways.

P. 2: § "*Experiments and mathematical model*" and § "*Wnt lignands*" make a long discussion about potential global biochemical gradient, but it is not clear what point the authors are trying to make. They should indicate here (rather than later) that other mechanisms might be at work and are worth exploring.

We changed our introduction and discussion to make the text clearer.

P. 3 « *We implement the mutual exclusion of these compartments inside cells and intercellular signalling using the differential adhesion properties of these compartments*. »: Justify why intracellular protein-protein interactions can be modelled with differential adhesion properties.

The contact term can be used to model adhesion properties, when specifying the interaction between domains belonging to different cells, or interface/contact energies when specifying interaction between domains belonging to the same cell. The use of the term adhesion in that sentence was incorrect and we changed it in the text.

P. 4 Eq 4: Choosing a constant cell-cell junction tension (rather than a variable one, which is more realistic) is a choice which is acceptable for the sake of simplification, but that should be explicitly mentioned and justified.

This simplification is now mentioned in the Model Overview section.

P. 5 « *We determined the exact values for each contact energy term by extensive parameter scans aimed at optimizing polarization for small systems.* »: It would be interesting to discuss the sensitivity of these parameters. It doesn't require a full analysis, but at least a qualitative discussion from experience which would help the reader to develop an intuition.

Added as Supplementary Figure S2.

P. 5 Eqs 5-6: Both the average of modulus and the modulus of average are interesting in the context of PCP and could be discussed. The authors seem to be interested only in the modulus of the average, which makes sense (I find that interesting in the context of the present question regarding long range ordering). However, according to Eq. 6; they seem to have measured the average of the modulus which makes sense for local ordering. Please clarify and justify.

There was a mistake in equation 6. We fixed it to be the modulus of the average.

P. 7 Fig. 2: There are too many arrows with different (and unclear) meanings. Suppress most of them, keep only the necessary ones, and provide a clear caption

We tried to minimize their use where possible and clearly indicate their meaning in the figure or legend.

P. 7 Fig.2 (and Supp Fig. 1): The choice of colors should be consistent. If subdomains are indicated by pink and light green, while schematics use either red-brown and dark green, it creates a confusion. Mutant cells could rather be evidenced using transparency.

Updated the colors of the mutant cells. For the Fz mutant, since the cells lack distal compartments and only have proximal compartments, they are shown in magenta (and vice-versa).

Up to P. 8 included the results are mostly expected ones. In particular: « *From these results, we conclude that the presence of the boundary signal in our model successfully biases the system to polarize along a preferred direction.* » Is this something that other models struggled with?

No. But the previous models did not systematically examine the effect of system size on global polarization and therefore could not determine the maximum range at which their models could polarize tissues.

P. 9 Fig. 3E caption: clarify « local polarization as a function of radial distance »

We described the method using which we calculated local polarization for both the boundary conditions in the Model Overview section.

P. 10, top: clarify and rewrite § "For the periodic" and § "We also examined".

2 typos: polalized, distancesup

P. 11 Fig. 4D: caption typo inidicated

Fixed.

Fig 4HIJ: add zooms of the cell and subcell compartments (possibly in the Supp)

The supplemental material now includes zoomed videos of those simulations where you can see both the cells and their compartments and the vectors.

P. 12, § "Instead": this is the main result of the paper, it is non-trivial, it should be more developed.

P. 12 Discussion: remove all redundancies with Introduction.

We rewrote the discussion and introduction to remove redundancies and to expand the discussion of the main results of the paper.

P. 13: Do daughter cells inherit PCP in a symmetric or asymmetric ways? Does that depend on the cell elongation?

The daughter cells always inherit the same amount of PCP domains, irrespective of the cell elongation and division plane. However, they always come out of the division in an unpolarized state, with all the domains mixed inside. We changed the text to make this more clear.

P. 13 bottom, § "Our results": overclaimed. Support or downtone the claims.

We rewrote the Discussion section.
