# Supplementary material for: Start small: A model for tissue-wide planar cell polarity without morphogens
Source: PLoS Comput Biol. 2026 Feb 12;22(2):e1013938. doi: 10.1371/journal.pcbi.1013938 (PMC12928585; doi:10.1371/journal.pcbi.1013938)
Supplement: Attachment — Submitted filename: responses_to_reviewers_PCP_final.pdf [file pcbi.1013938.s026.pdf]

Reviewer #1: The authors have done an excellent job responding to reviewers comments and suggestions. The addition of a new section comparing autonomous vs non-autonomous PCP mechanisms is timely and strengthens the paper. The additional context and model predictions added to the discussion also improve readability. Overall this is a valuable and well executed study that with have substantial impact on the polarity field.

Reviewer #2: I appreciate the care taken in addressing the reviewer comments, which has greatly improved the manuscript.

I have only two remaining (relatively minor) comments:

- It seems as if many of the newly added supplementary figures are only mentioned the first time in the discussion, which reads a bit odd given that the discussion is normally not intended to present new data. I would suggest to integrate these with the results section.

All supplementary figures are now referenced in the Results section.

- RE setting the time scale of the CPM: I understand the author's comment that this is difficult because no experimental measurement of polarization time is available, so I'll leave the choice up to them. However, one of the other reviewers mentioned the same issue so I think it might be worth making an attempt. I think that even if polarization time is not experimentally measured, it is probably possible to provide a (very rough!) estimate of at least the order of magnitude of this polarization time. Even if you still measure time in MCS, you could then at least test in what order of magnitude the tissue polarization time would end up, and to what extent that is realistic - I don't think the estimate needs to be perfect to give at least *some* indication that would massively help with interpretation here and as such improve the paper's impact. However, given the large amount of additional work the authors already did, I'll leave the choice up to them (it's not strictly necessary for the paper but would make it stronger if possible).

We set the time scale of the simulations in terms of the time of the time it takes for a single cell in the absence of neighbours to polarize. We define this time as ***polarization time (-tau-)*** *and use this to get an idea of the order of magnitude of the time taken by systems with and without proliferation to polarize. To this end, we added the rescaled time axes to the graphs in Main Figures 1 and 4 (added as Supplementary Figure S3) and Supplementary Figure 1. With this modification, we can see that it takes about 1500 times more time to polarize a system of 4x4 cells (with periodic boundary conditions) as compared to the time it takes for a single cell to polarize.*

Reviewer #3: The revision is adequate
